# Determination of the protective effects of Hua‐Zhuo‐Jie‐Du in chronic atrophic gastritis by regulating intestinal microbiota and metabolites: combination of liquid chromatograph mass spectrometer metabolic profiling and 16S rRNA gene sequencing

**DOI:** 10.1186/s13020-021-00445-y

**Published:** 2021-05-01

**Authors:** Pingping Zhou, Xinyu Hao, Yu Liu, Zeqi Yang, Miaochan Xu, Shaowei Liu, Shixiong Zhang, Tianxiao Yang, Xiaomei Wang, Yangang Wang

**Affiliations:** 1 Hebei University of Chinese Medicine, Xinshi South Road No 326, Qiaoxi District, Hebei 050091 Shijiazhuang, China; 2 Beijing University of Chinese Medicine Third Affiliated Hospital, Anwai Xiaoguan Street No. 51, Chaoyang District, 100029 Beijing, China

**Keywords:** Hua‐Zhuo‐Jie‐Du, Chronic atrophic gastritis, LC–MS, 16S rRNA gene sequencing

## Abstract

**Background:**

Hua-Zhuo-Jie-Du (HZJD), a Chinese herbal prescription consisting of 11 herbs, is commonly used in China to treat chronic atrophic gastritis (CAG). We aimed to determine the effect of HZJD on the microbiome-associated metabolic changes in CAG rats.

**Methods:**

The CAG rat models were induced by 1-methyl-3-nitro-1-nitrosoguanidine (MNNG) combined with irregular fasting and 2% sodium salicylate, which was intragastrically administrated in fasted animals for 24 weeks. The CAG rats in the Chinese medicine (CM) group were administered a daily dose of 14.81 g/kg/day HZJD, and the vitacoenzyme (V) group were administered a daily dose of 0.08 g/kg/day vitacoenzyme. All animals were treated for 10 consecutive weeks, consecutively. Hematoxylin and eosin (H&E) staining was used to assess the histopathological changes in the gastric tissues. An integrated approach based on liquid chromatograph mass spectrometer (LC-MS) metabolic profiling combined with 16S rRNA gene sequencing was carried out to assess the effects of HZJD on CAG rats. Spearman analysis was used to calculate the correlation coefficient between the different intestinal microbiota and the metabolites.

**Results:**

The H&E results indicated that HZJD could improve the pathological condition of CAG rats. The LC–MS results indicated that HZJD could significantly improve 21 gastric mucosal tissue perturbed metabolites in CAG rats; the affected metabolites were found to be involved in multiple metabolic pathways, such as the central carbon metabolism in cancer. The results of 16S rRNA gene sequencing indicated that HZJD could regulate the diversity, microbial composition, and abundance of the intestinal microbiota of CAG rats. Following HZJD treatment, the relative abundance of *Turicibacter* was increased, and the relative abundance of *Desulfococcus* and *Escherichia* were decreased in the CM group when compared with the M group. Spearman analysis revealed that perturbed intestinal microbes had a strong correlation with differential metabolites, *Escherichia* exhibited a negative correlation with l-Leucine, *Turicibacter* was negatively correlated with urea, and *Desulfococcus* exhibited a positive correlation with trimethylamine, and a negative correlation with choline.

**Conclusions:**

HZJD could protect CAG by regulating intestinal microbiota and its metabolites.

## Background

Chronic atrophic gastritis (CAG) is a disease involving chronic inflammation of the gastric mucosa and is considered to be an important precursor of gastric cancer. Previous studies have shown that CAG is the most common cause of cancer-related death and is ranked second among other cancer-related causes of death worldwide [1, 2]. CAG has increased in incidence because of various factors, such as eating habits, smoking [3], and microbial infections [4]. The treatment of CAG mainly involves protecting the gastric mucosa, vitamin C supplementation, endoscopic minimally invasive treatment and eradication of *Helicobacter pylori* [5–7]. These treatments, however, are often accompanied by adverse reactions [8]; therefore, complementary and alternative intervention to treat CAG are urgently required. At present, traditional Chinese medicine (TCM) has been shown to be one of the most promising methods for the treatment of CAG [9–12].

In TCM, Hua-Zhuo-Jie-Du (HZJD) is a Chinese formula that is widely used in the treatment of CAG based on the TCM theories of clearing away heat and dampness, and removing turbidity and detoxification. HZJD consists of 11 herbs including *Artemisia capillaris* Thunb. (Yinchen), *Scutellaria baicalensis* Georgi (Huangqin), *Coptis chinensis* Franch. (Huanglian), *Scutellaria barbata* D.Don (Banzhilian), *Scleromitrion diffusum* (Willd.) R.J.Wang (Baihuasheshecao), *Isatis tinctoria* L. (Banlangen), *Pogostemon cablin* (Blanco) Benth. (Guanghuoxiang), *Lobelia chinensis* Lour. (Banbianlian), *Sophora flavescens* Aiton (Kushen), Eupatorium fortunei Turcz. (Peilan) and *Gynostemma pentaphyllum* (Thunb.) Makino (Jiaogulan). Previous studies have reported on the possible mechanism of HZJD in the treatment of CAG, which is mainly suggested to be due to the regulation of cell proliferation and apoptosis [13, 14]. In a clinical study, we compared the clinical efficacy, gastroscopic efficacy and pathological efficacy of HZJD and ALaTanWuWeiWan for gastric precancerous lesions, and found that the efficacy of HZJD was better than the control group (*P* < 0.05), the mechanism was thought to be associated with the decreased expression of HIF-1α, and VEGF and the increased expression of PTEN [15]. TCM is a coordinated system for the treatment of multitarget and multicomponent diseases. In a network pharmacology study, we constructed the “herb-compound-target-disease” network diagrams, and found that 156 active ingredients in HZJD had common targets with CAG [13]. Metabolomics is a systems biology approach that comprehensively describes the pharmacological action of drugs and the association between disease processes and specific biological pathways. Metabolomics has been increasingly applied to establish the therapeutic effect of TCM [16, 17] and has the potential to determine the novel regulatory mechanism of HZJD. Liquid chromatograph mass spectrometer (LC-MS) is an analytical method that is suitable for the separation and quantification of hard-to-volatile compounds or compounds with poor thermal stability. At present, the identification of metabolites has been widely used in metabolomics research [18].

Approximately 100 trillion gut microbes exist in the human body. Specifically, the gut microbiota are composed of large number of different bacteria that produce various metabolites and participate in important metabolic functions, such as membraneand energy metabolism [19, 20]. Accumulating evidence has shown that the gut microbiota contribute to the regulation of gastrointestinal function [21]. Previous studies have shown that the abundance of bacteria in patients with CAG is increased with the reduced secretion of gastric acid and that the changes in intestinal microbiota contribute to the progression from intestinal metaplasia (IM) to gastric cancer [22–24]. However, the association between the metabolic phenotype and intestinal microbiota in CAG and the changes noted during HZJD treatment of CAG remain unclear.

The present study investigated the effect of HZJD on the microbiome-associated metabolic changes in CAG rats by employing an LC–MS-based metabolomics method coupled with 16S rRNA gene sequencing. To the best of our knowledge, this is the first study to examine the effect of HZJD on the intestinal microbiota and metabolites of CAG rats.

## Materials and methods

### Preparation of HZJD

We prepared HZJD using the following procedure: *Artemisia capillaris* Thunb. (Yinchen) 15 g, *Scutellaria baicalensis* Georgi (Huangqin) 12 g, *Coptis chinensis* Franch. (Huanglian) 12 g, *Scutellaria barbata* D.Don (Banzhilian) 15 g, *Scleromitrion diffusum* (Willd) R.J.Wang (Baihuasheshecao) 15 g, *Isatis tinctoria* L. (Banlangen) 15 g, *Pogostemon cablin* (Blanco) Benth. (Guanghuoxiang) 9 g, *Lobelia chinensis* Lour. (Banbianlian) 15 g, *Sophora flavescens* Aiton (Kushen) 10 g, *Eupatorium fortunei* Turcz. (Peilan) 9 g and *Gynostemma pentaphyllum* (Thunb.) Makino (Jiaogulan) 15 g were mixed together, and then diluted with distilled water to a concentration of 0.325 g/ml. The formula granules were provided by E-FONG Pharmaceutical Co., Ltd (Guangdong, China). High performance liquid chromatography (HPLC) showed that the preparation method of HZJD was both stable and feasible.

### Animals and treatment

Sprague Dawley (SD) rats (body weight, 150–180 g), were obtained from Liaoning Changsheng Biotechnology Co., Ltd (Liaoning, China). All of the processes related to animal care and use were approved by the Institutional Animal Care and Use Committee of the Hebei University of Chinese Medicine (DWLL2019012). The rats were maintained in a temperature-controlled environment (24 ± 4 °C) with a 12/12 h light-dark cycle with free access to food and water. After 1 week of adaptation, eight rats were randomly selected as the normal group and the remaining rats were used to establish a CAG rat model. The replication of the CAG rat model was performed according to previous studies [11, 12, 25], with minor modifications. Briefly, the normal group had free access to clean water and a normal diet. The CAG rat models were induced by 1-methyl-3-nitro-1-nitrosoguanidine (MNNG, 200 µg/mL; Tokyo Chemical Industry Co., Ltd, Tokyo, Japan) combined with irregular fasting and 2% sodium salicylate (Sangon Biotech Co., Ltd, Shanghai, China) was intragastrically administrated to fasted animals for 24 weeks. Two rats were selected randomly from the model group and were humanely killed by intraperitoneal injection of sodium pentobarbital (140 mg/kg). The stomach was obtained for pathological examination to confirm that the CAG model has been successfully establishied. Subsequently, the CAG rat models were randomly divided into a model group (M group), a Chinese medicine group (CM group) and a vitacoenzyme group (V group) (n = 8 rats per group). Rats in the CM group were administered a daily dose of 14.81 g/kg/day HZJD, rats in the V group were administered a daily dose of 0.08 g/kg/day vitacoenzyme.

### Sample collection and preparation

Following fasting without water for 24 h, the SD rats were humanely killed with sodium pentobarbital (140 mg/kg i.p.) and the gastric tissues and feces were collected. Feces were stored at − 80 °C until required for further analysis by 16S rRNA gene sequencing. The stomach tissues were divided into two parts on an ice tray; one part was immersed in paraformaldehyde (Servicebio, Wuhan, China) for histological evaluation, and the other part was quickly stored at − 80 °C until required for analysis by LC–MS.

### Hematoxylin and eosin staining

We conducted hematoxylin and eosin (H&E) staining to observe the histological changes in the tissues. Tissues were immersed in paraformaldehyde for 24 h and embedded in paraffin wax blocks. Then, the paraffin-embedded tissues were cut into two consecutive slices (4 μm thickness), and the slices were placed on a glass slide for incubation, conventional dewaxing using xylene and dehydration via gradient alcohol. We performed H&E staining and observed the histopathology of the gastric mucosa under an optical microscope (Nikon Group Companies, Japan, model: ECLIPSE NI-U).

### Liquid chromatography quadrupole time-of-flight mass spectrometer conditions and mass spectrometry

We conducted the analyses using UHPLC (1290 Infinity LC, Agilent Technologies) coupled to a quadrupole time-of-flight mass spectrometer (AB SciexTripleTOF 6600; Shanghai Applied Protein Technology Co., Ltd).

We performed hydrop interaction liquid chromatography (HILIC) separation using a 2.1 mm × 100 mm 1.7 μm column (ACQUIY UPLC BEH; Waters Corporation, Milford, MA, USA) to analyze the samples. In both positive and negative electrospray ionization (ESI) modes, the mobile phase contained solvents A (25 mM ammonium acetate and 25 mM ammonium hydroxide) and B (acetonitrile). The gradient used was as follows: 85% B at 1 min, linear decrease to 65% at 11 min, 0.1 min decrease to 40% and constant solvent composition for 4 min, 0.1 min increase to 85% and 5 min of equilibration time.

We performed reversed-phase liquid chromatography (RPLC) using a 2.1 mm × 100 mm 1.8 μm column (ACQUIY UPLC HSS T3; Waters Corporation). In the ESI positive mode, the mobile phase was as follows: A, water and 0.1% formic acid; B, acetonitrile and 0.1% formic acid. In the ESI negative mode, the mobile phase contained A, 0.5 mM ammonium fluoride and B, acetonitrile. The gradient was as follows: 1% B for 1.5 min, linear increase to 99% up to 11.5 min, constant solvent composition for 3.5 min, decrease to 1% for 0.1 min and 3.4 min of equilibration. The gradient flow rate was 0.3 mL/min and the column temperature was maintained at 25 °C. Each sample injection contained 2 µL.

During tandem mass spectrometry MS/MS analysis, the ESI source conditions were set as follows: The ion source Gas1 and Gas2 were 60 and the curtain gas was 30. The source temperature was set at 600 ℃ and the ion spray voltage floating was set at 5500 V. In the MS-only acquisition mode, the instrument was set to acquire a m/z range of 60–1000 Da and the TOFMS scan accumulation time was set to 0.20 s/spectra. The MS/MS automatic acquisition was set to acquire a m/z range of 25–1,000 Da and the product ion scan accumulation time was set to 0.05 s/spectra. The product ion scan adopted information dependent acquisition and the high sensitivity-mode was selected. The parameter settings were as follows: fixed collision energy 35 v and 15 eV and dimming potential 60 v (+) and 60 v (−). We performed the isotopes within 4 Da and monitored 10 candidate ionsper cycle.

### 16S rRNA gene sequencing analysis

We isolated total DNA from rat fecal pellets using the cetyl trimethyl ammonium bromide/sodium dodecyl sulfate (CTAB/SDS) method. We selected the V3–V4 region of 16S rRNA genes using specific primers with the following barcode: 341 F-806R. The polymerase chain reaction (PCR) products were detected by 2% agarose gel electrophoresis and the target fragments were cut and recovered. The AxyPrepDNA (Axygen Scientific Inc. USA) gel recovery kit was used.

We detected the PCR amplification products by the QuantiFluor™-ST blue fluorescence quantitative system (Promega Corporation, Madison, WI, USA). The corresponding proportions were mixed according to sequencing volume requirements of each sample. Subsequently, we used the Ultra™ DNA Library Prep Kit (NEB, USA) for library construction. The library was sequenced on a computer following quality-check performed by agilent bioanalyzer 2100 (Agilent, Palo Alto, CA, USA) and qubit.

We performed sequence analyses using the UPARSE software package with the UPARSE-OTU and UPARSE-out ref algorithms. In-house perl scripts were used to analyze the alpha (within samples) and beta (among samples) diversity values. We assigned sequences with in-house perl to the same operational taxonomic units (OTUs).

### Statistical analysis

One way ANOVA (GraphPad Prism 8) was used to analyze the changes in metabolites and abundance of the intestinal microbiota among different groups, *P* < 0.05 was considered statistically different. Date were expressed as means ± standard deviation.

## Results

### Morphological changes of the gastric mucosa

We conducted histological examination of gastric mucosa samples derived from rats. We employed H&E staining to evaluate the pathological changes. In the N group, visual observation indicated that the gastric mucosa was smooth and mainly red and that the gastric folds were well-arranged. H&E staining showed that the gastric mucosal epithelial cells and the glands had well preserved integrity and were arranged regularly. We did not observe any expansion or hyperemia or any inflammatory cellular infiltration in the submucosa and muscularis.

In contrast to these findings, the gastric mucosa of CAG rats exhibited a white color, reduced number of gastric folds and visible white nodules. H&E staining demonstrated disorders of gastric mucosal glands in the CAG rats accompanied by inflammatory cellular infiltration and muscularis. Taken together, these results illustrated that the CAG rat model was established successfully.

**Fig. 1:**
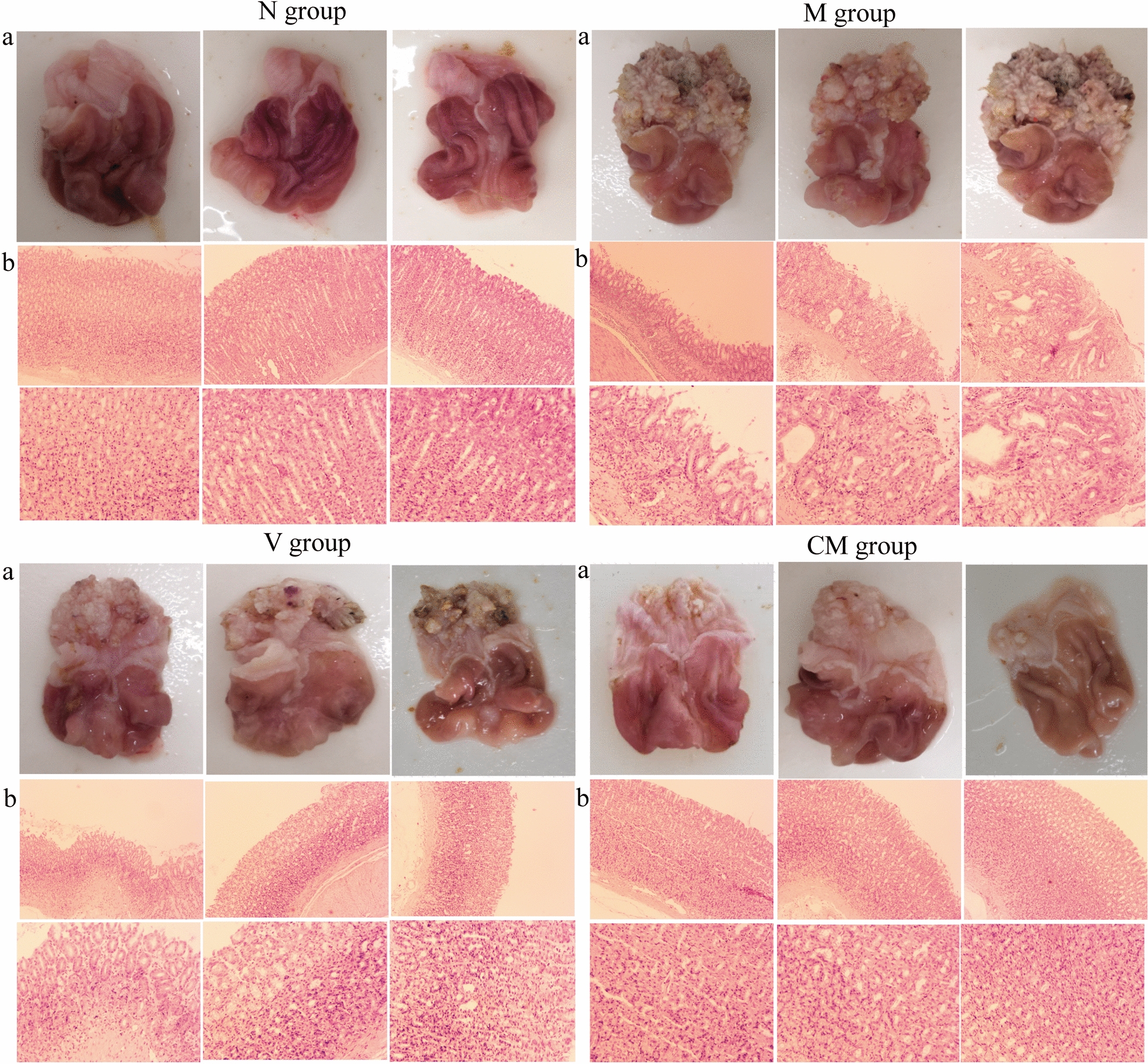
Effects of Hua-Zhuo-Jie-Du (HZJD) on gastric histological changes. Representative images of different groups. **a** The gastric mucosa under visual observation. **b** The pathological changes in the gastric mucosa under H&E staining. H&E ×40 for the second row and H&E ×100 for the bottom row

The gastric mucosa morphology was significantly improved in the CM group compared with the M group. The gastric mucosa of rats in CM group was smooth and exhibited a red color with a small percentage of white nodules. H&E staining indicated that the glands were neatly arranged and a complete glandular tube structure was clearly observed. No abnormal thickening of the muscularis mucosa was noted. In the V group, the gastric mucosa was mainly white, but with obvious white nodules. H&E staining showed that the glands were disordered, inflammatory cell infiltration was visible, and there was no clear pathological improvement. On the basis of these pathological manifestations, we considered HZJD to be effective for the treatment of CAG and found that it was better than vitacoenzyme treatment (Fig. 1).

## LC**–**MS method validation

**Fig. 2:**
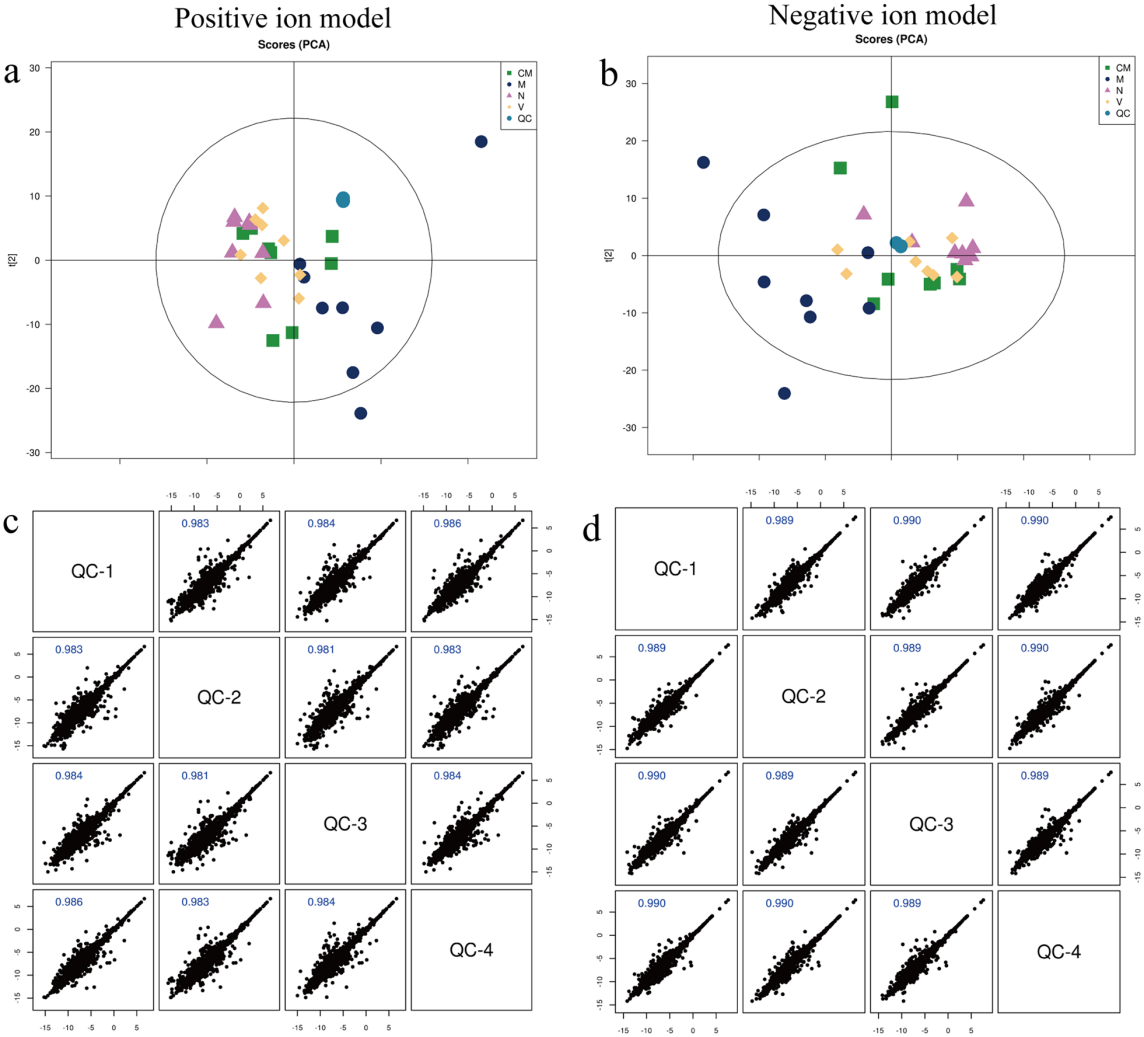
Quality control (QC) chart for samples. **a**,** b** Principal component analysis (PCA) score charts in the positive and negative ion modes, different graphs represent different samples. Chinese medicine (CM) group samples = Green square, model (M) group samples = Dark blue dots, normal (N) group samples = Purple triangle, vitacoenzyme (V) group samples = Yellow rhombus, and QC samples = Light blue dots. **c**,** d** QC sample correlation map for the positive and negative ion modes

We used XCMS software (SIMCA-P 14.1, Umetrics, Umea, Sweden) to extract the metabolite ion peaks of all samples. A total of 13,150 peaks were obtained in the positive ion mode, and 9173 peaks were obtained in the negative ion mode. We obtained the principal component analysis (PCA) model by pareto-scaling conversion of all peaks. As shown in Fig. 2a, b, the quality control (QC) samples were closely clustered in the positive and negative ion modes, which proved that the experiment had good repeatability. We performed pearson correlation analysis on the QC samples. The abscissa and ordinate marked the logarithm of the intensity value. Generally, a correlation coefficient greater than 0.9 indicated a good correlation. As shown in Fig. 2c, d, all correlation coefficients were greater than 0.9, indicating that the instrument analysis system was stable and the data could be used for subsequent analysis.

### HZJD-treated gastric tissue metabolic profiling analysis

**Fig. 3:**
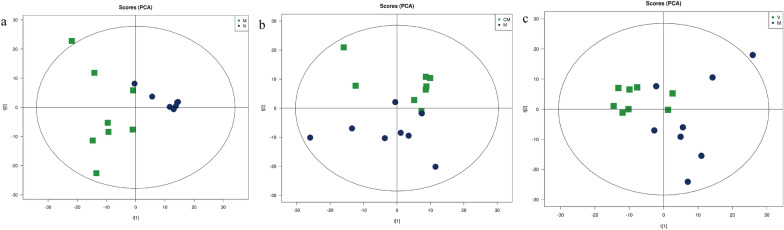
PCA score charts for the positive ion mode. **a** M and N groups. M = Green square and N = Dark blue dots. **b** M and CM groups. CM = Green square and M = Dark blue dots. **c** M and V groups. V = Green square and M = Dark blue dots

**Fig. 4:**
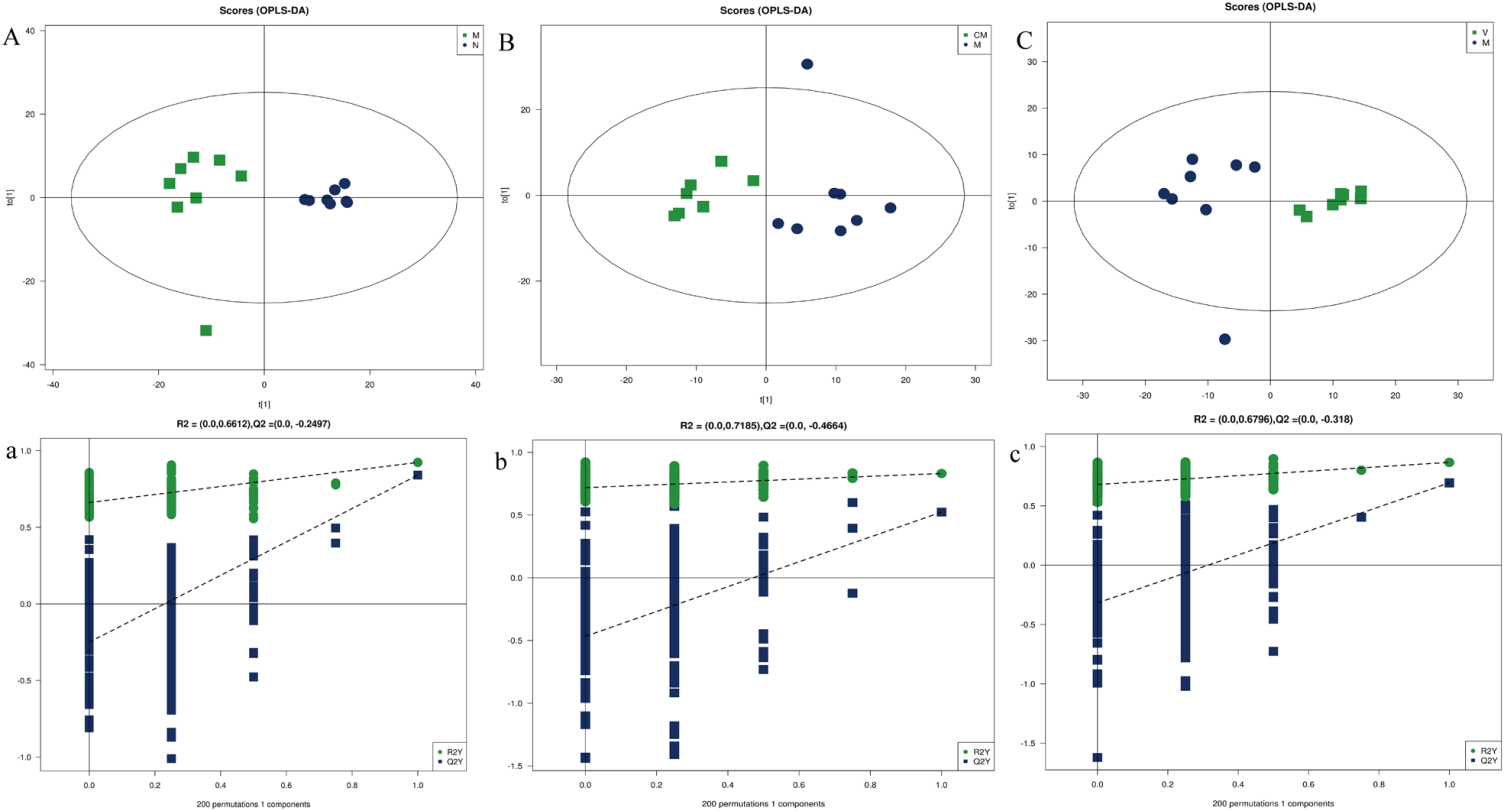
Orthogonal partial least square-discriminate analysis (OPLS-DA) score charts and cross-validation test in the positive ion mode. **A**, **a** OPLS-DA score graph and mode cross-validation graph for the M and N groups. M = Green square and N = Dark blue dots. **B**,** b** OPLS-DA score graph and mode cross-validation graph for the CM and M groups. CM = Green square and M = Dark blue dots. **C**, **c** OPLS-DA score graph and mode cross-validation graph for the V and M groups. V = Green square and M = Dark blue dots

**Fig. 5:**
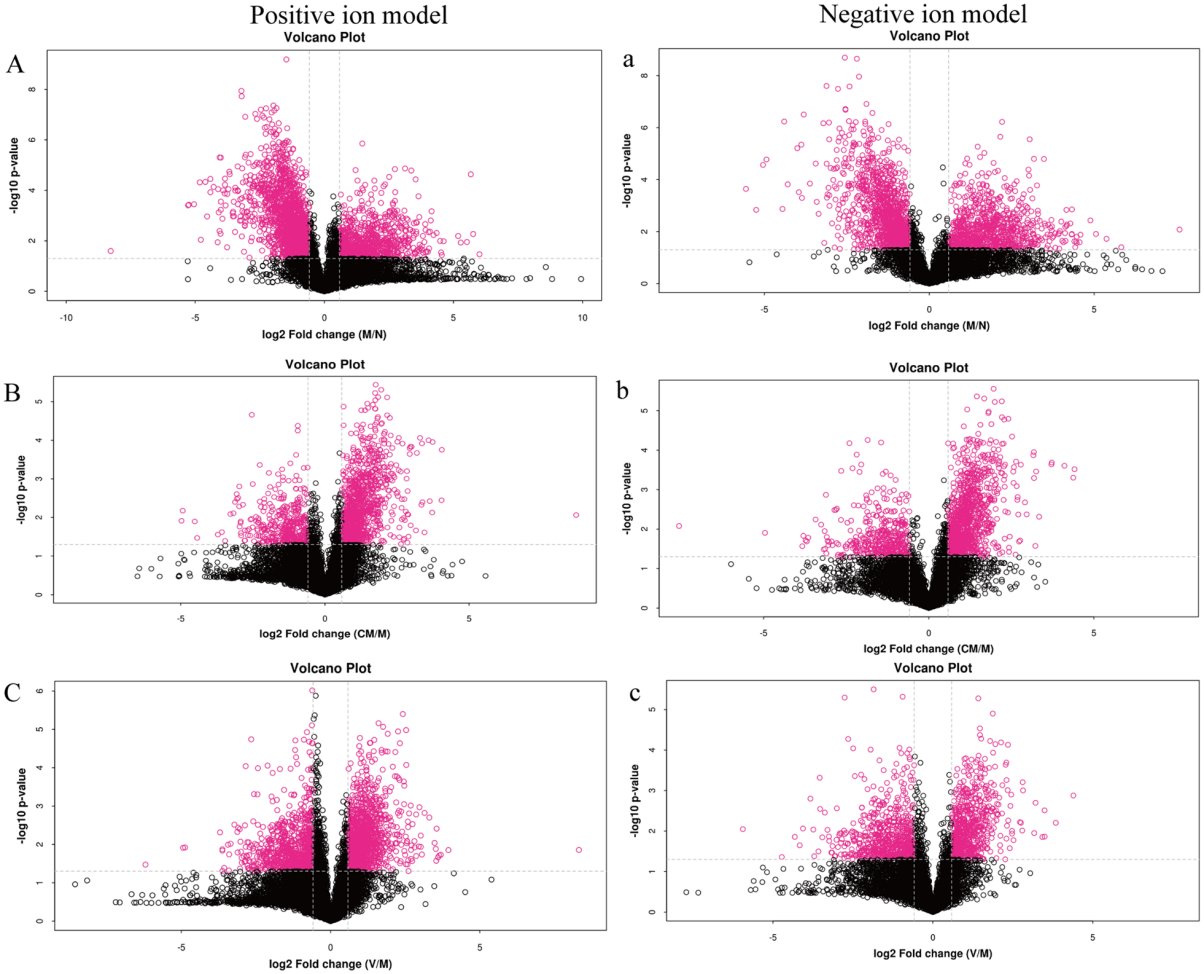
Volcano map based on fold change (FC) analysis and t test. **A**, **a** M and N volcano diagrams in the positive and negative ion modes. M = Left, N = Right. **B**,** b** CM and M volcano diagrams in the positive and negative ion model. CM = Left, M = Right. **C**, ** c** V and M volcano diagrams in the positive and negative ion modes. V = Left, M = Right. The red dots in the figures are differential metabolites with FC > 1.5 and *P* < 0.05. Black dots represent metabolites with no significant difference

The PCA model reflected the variability between groups and within groups. The PCA model (Fig. 2a, b) showed that the M group had two samples that overlapped with the HZJD group and the V group. To further observe the changes in the metabolites, we used PCA analysis to analyze the N and M groups, the M and CM groups, and the M and V groups. As shown in Fig. 3, the N and M group had one sample overlapping, and the CM and V group had two samples overlap with the M group, but the overall trend of distinction was clear, which indicated that the metabolites of the CAG rats had changed. Subsequently, the orthogonal partial least square discriminate analysis (OPLS-DA) model was constructed, and cross-validation proved that the model was reliable (Fig. 4). In the OPLS-DA model, a distinct separation was presented between the M and the N groups, the M and CM groups, and the M and V groups, suggesting that the concentration levels of the metabolites in the CAG rat models were significantly changed. Next, we used fold change (FC) analysis and t-test to further analyze the changes between the two groups of samples. As shown in Fig. 5, the M group and the N group, the M group and the CM group, and the M group and the V group were significantly separated in the positive and negative ion modes.

**Fig. 6:**
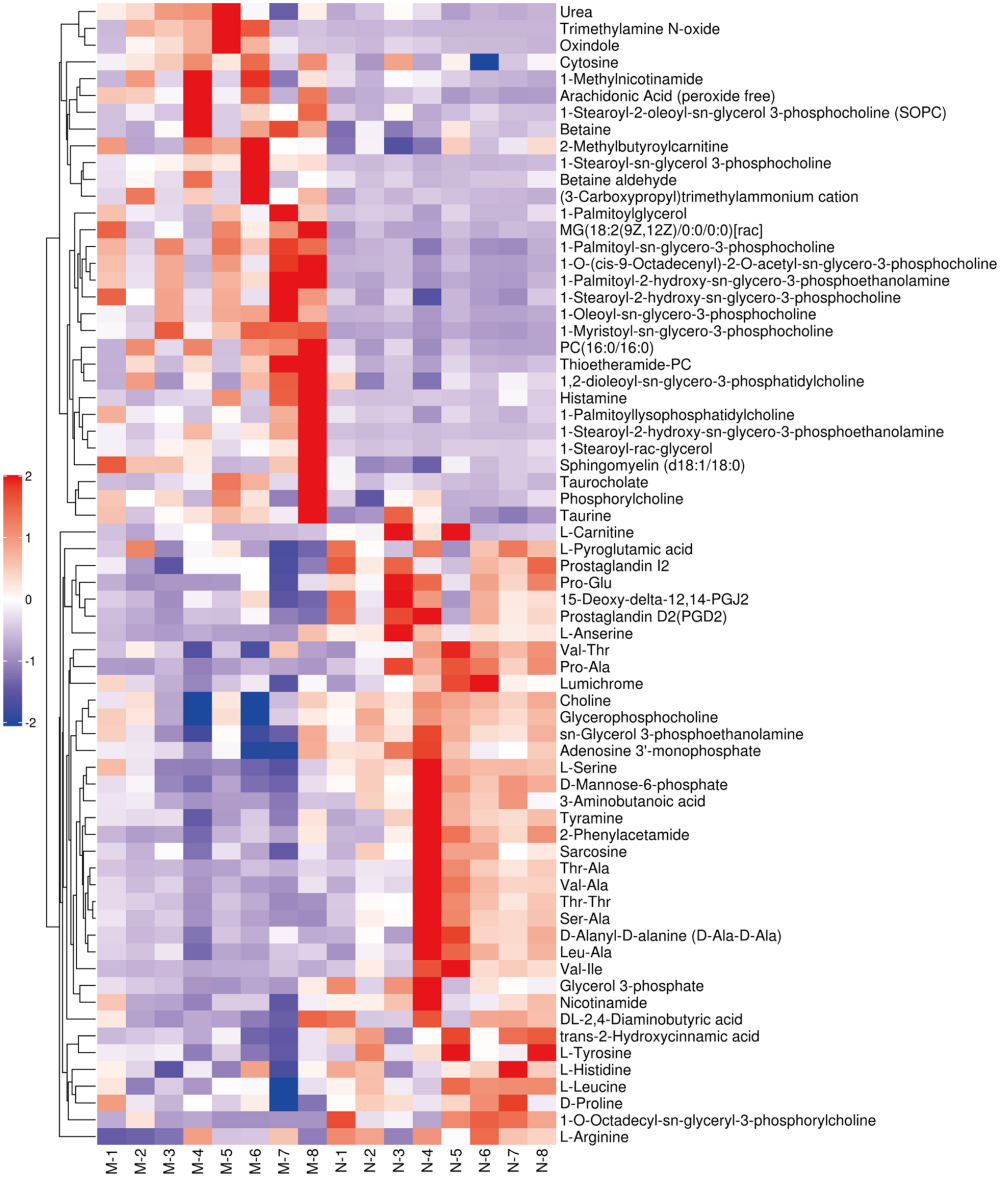
Differential metabolite hierarchical clustering diagram. The ordinate represents the metabolites that are significantly differently expressed, and the abscissa is the sample information. Red represents significantly up-regulated metabolites, blue represents significantly down-regulated metabolites, and the gray part represents no quantitative information on the metabolite

Based on the difference between the N and M groups of the OPLS-DA model, variable values were set at variable importance for the projection (VIP) > 1 and *P* < 0.05 to assess the differential metabolites that lead to the occurrence of CAG. The biomarkers were identified according to their structures in the Human Metabolome Database (HMDB; http://www.hmdb.ca/). Notably, we identified 68 metabolites in the current study. To evaluate the rationality of metabolites, and to intuitively display the relationship between samples and the differences in the expression of metabolites in different samples, we used the level of expression of metabolites to perform hierarchical clustering of each group of samples; this allowed us to accurately screen marker metabolites and study the changes in related metabolic processes. As shown in Fig. 6, in this study, compared with the N group, the metabolites of CAG rats changed significantly. The changes in these metabolites may be related to the occurrence of CAG.

**Fig. 7:**
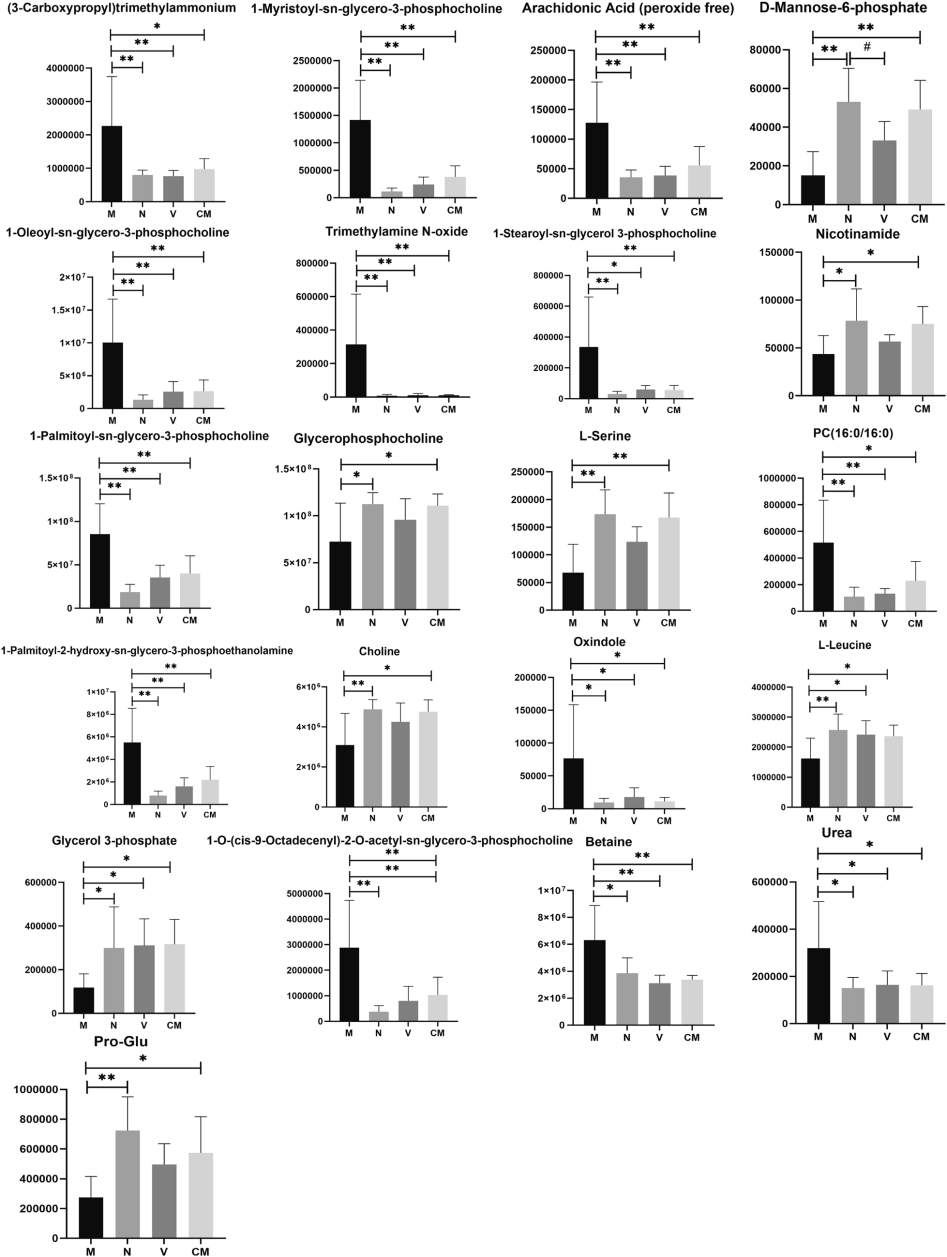
Effect of HZJD on differential metabolites. Compared with the M group, **p < 0.01; *p < 0.05. Compared with the N group, ^##^*p* < 0.01; ^#^*p* < 0.05

**Fig. 8:**
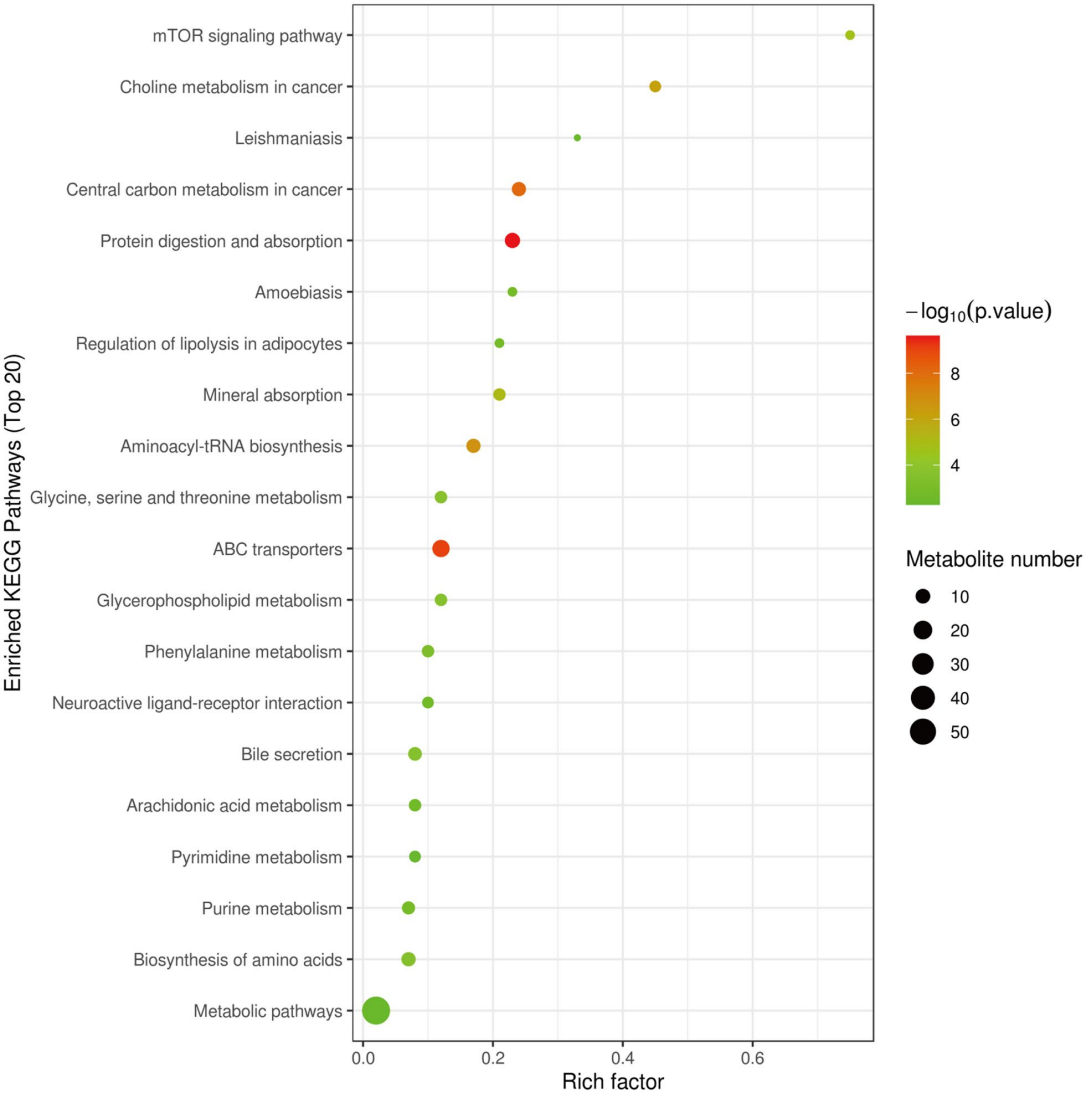
Kyoto encyclopedia of genes and genomes (KEGG) pathway enrichment analysis of differentially expressed metabolites

Following HZJD treatment, 21 metabolites showed a recovery trend (Fig. 7). Based on the differential metabolites, we employed the Kyoto Encyclopedia of Genes and Genomes (KEGG) (http://www.kegg.jp/) to explore the most relevant pathways. We used Fisher’s exact test to analyze the significance level of metabolite enrichment of each pathway to determine the metabolic and signal transduction pathways that were significantly affected. The top 20 affected transduction pathways are described in Fig. 8 and include the mTOR signaling pathway and choline metabolism in cancer.

### HZJD-induced changes in the gut microbiome

**Fig. 9:**
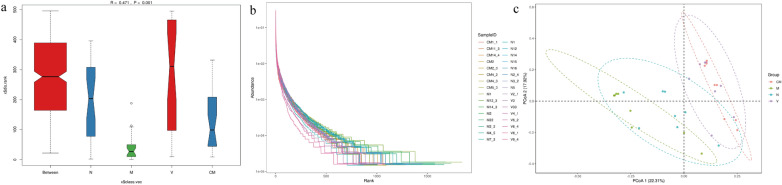
**a** Analysis of similarities results. Between is the result of the comparison between groups, and others are the results in their respective groups. In the present study, R = 0.471, *P* = 0.001. **b** The OTU rank curve of the intestinal microbiota in rats. Different colored curves represent different samples. The abundance of species is reflected by the width of the curve. The higher the abundance of the species, the larger the range of the curve on the horizontal axis; the shape (smoothness) of the curve reflects the uniformity of the species in the sample, and the smoother the curve, the more uniform the distribution of species. **c** The composition of the rat intestinal community among different groups. Different colors represent different samples. CM = Pink, M = Green, N = blue, and V = purple

To judge whether the grouping was meaningful, we used analysis of similarities to test whether the difference between the groups was greater than the difference within the group. The R value was between (− 1, 1), and the R > 0, indicating that the difference between the groups was significant. The R < 0 indicates that the difference within the group was greater than the difference between the groups. The reliability of the statistical analysis is expressed by *P* value, and *P* < 0.05 indicates statistical significance. In the present study, R = 0.471, *P* = 0.001 (Fig. 9a), which indicating that the difference between groups was greater than the difference within groups, and that the grouping is reasonable. The sequences were clustered into OTUs with 97% identity. The OUTs in the M group were significantly different, and the rank-abundance of the slope of the M group was gentlerand spread wider on the horizontal axis compared with the N group. Following HZJD and vitacoenzyme treatment, the abundance and uniformity of species had changed significantly, and the N, HZJD, and V groups showed a consistent trend (Fig. 9b). We next sought to assess whether the microbiota composition differed among the N, M, CM and V groups. To assess this, we conducted principal coordinates analysis (PCoA) on the unweighted uniFrac distance to analyze the difference in gut microbial community structures. As shown in Fig. 9c, there was no obvious separation between the N and M groups, the M group was clearly separated from the CM and V groups, and the CM group could not be clearly separated from the V group. These findings indicated that both HZJD and vitacoenzyme influenced the gut microbial composition and that oral administration of HZJD ameliorated the gut microbiome of CAG rats to a certain extent.

### HZJD altered the abundance of the gut microbiome

**Fig. 10:**
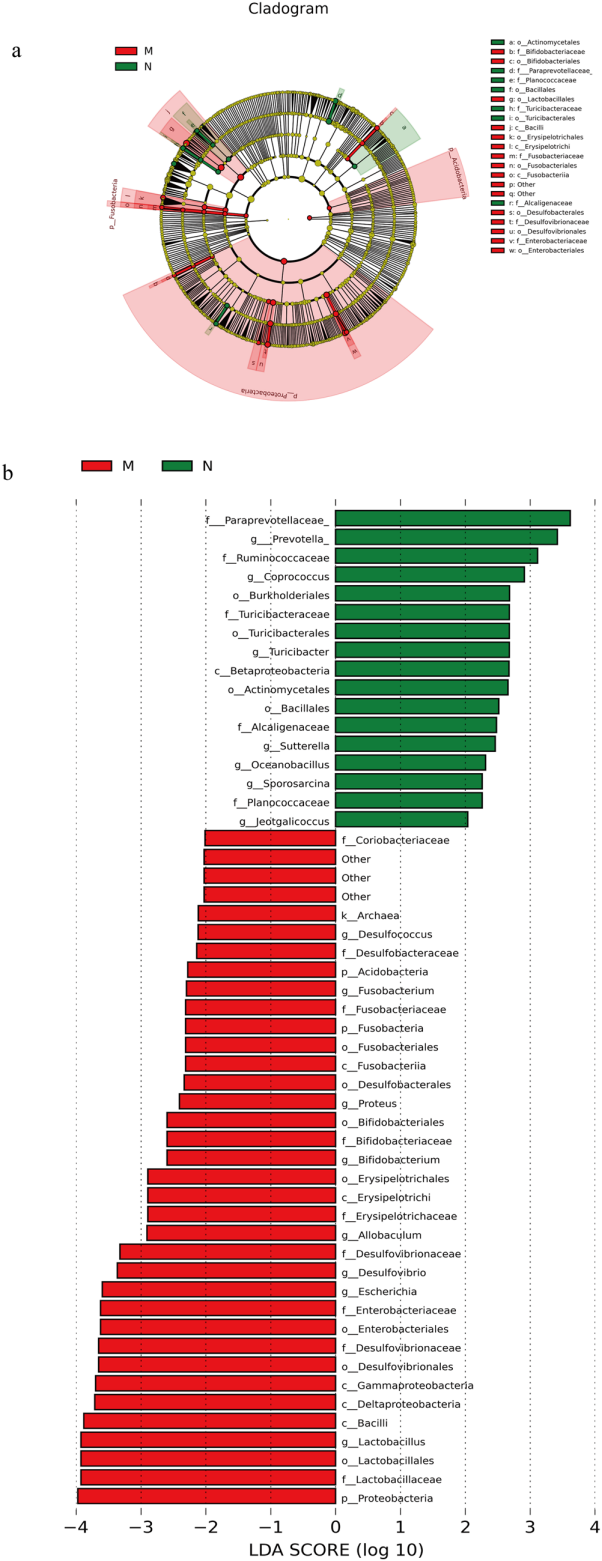
Differences in the microbiota composition between the N and M groups with LEfSe (LDA > 2). **a** Cladogram. Each circle represents the classified level from the phylum to the species. The diameter of the small circle is proportional to the relative abundance of gut microbiota. **b** Histogram. M = Red, representing the intestinal microbiota significantly enriched in the mode group. N = Green, representing the intestinal microbiota significantly enriched in the normal group

**Fig. 11:**
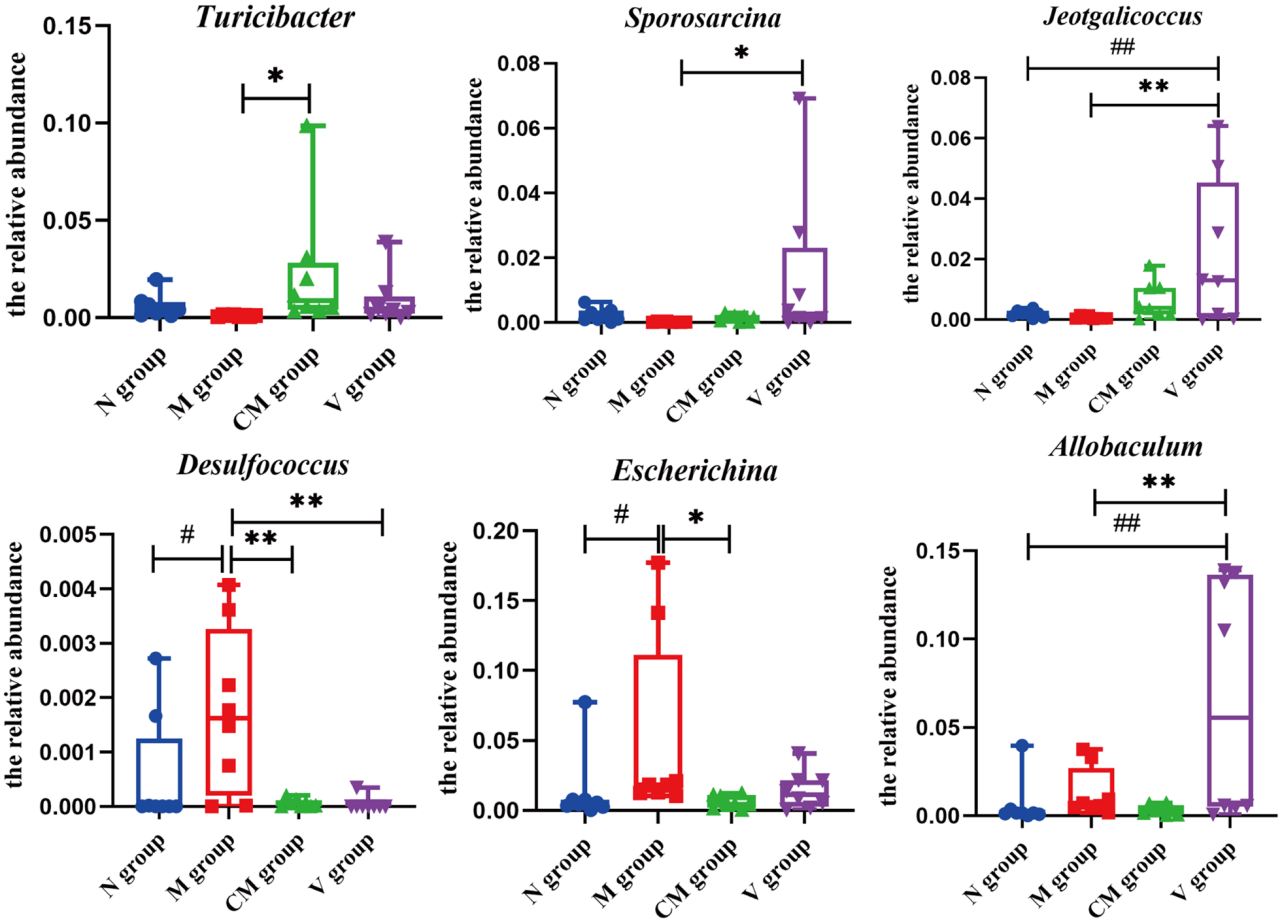
Effect of HZJD on the relative abundance of intestinal microbiota at the genus level. Compared with the M group, **p < 0.01; *p < 0.05. Compared with the N group, ^##^*p* < 0.01; ^#^*p* < 0.05

To analyze the intestinal microbiota that caused this difference in abundance, we employed linear discriminant analysis (LDA) and effect site linear discriminant analysis effect size (LEfSe) to explore the differences between the N and M groups through an analysis of taxon abundance in the gut microbiota. A cladogram was obtained by the LEfSe method, and Fig. 10a shows the different microbial communities of each group at different levels. Fifteen different genera were identified in the intestinal microbiota of the N and M groups (LEfSe LDA > 2 and *P* < 0.05). *Prevotella, Coprococcus, Turicibacter, Sutterella, Oceanobacillus, Sporosarcina*, and *Jeotgalicoccus* were increased in the N group, whereas *Desulfococcus, Fusobacterium, Proteus, Bifidobacterium, Allobaculum, Desulfovibrio, Escherichia*, and *Lactobacillus* were increased in the M group (Fig. 10b). Following HZJD and vitacoenzyme treatment, compared with the M group, the relative abundance of *Turicibacter*, *Sporosarcina* and *Jeotgalicoccus* increased; the abundance of *Desulfococcus*, *Escherichia*, and *Allobaculum* decreased; and the abundance of *Turicibacter*, *Desulfococcus* and *Escherichia* had statistically significant changes in the CM group. These results indicated that HZJD contributed to the prevention of the microbiota changes noted in the CAG rats (Fig. 11).

### Correlation of the gut microbiota and gastric tissues metabolic phenotype in CAG rats

We conducted further analysis to explore the functional correlation between the metabolite levels and the microbial identity. First, we organized the relative abundance of the bacterial populations with significant differences at the genus level (LEfSe LDA > 2 and *P* < 0.05) obtained by 16S rRNA gene sequencing analysis, and the significantly different metabolites obtained by LC–MS analysis (VIP > 1 and *P* < 0.05) in a table as input files for subsequent analysis. Taking into account the non-normal distribution of the original data, we used the spearman analysis method to calculate the correlation coefficient between the significantly different intestinal microbiota and the significantly different metabolites in the experimental samples.

**Fig. 12:**
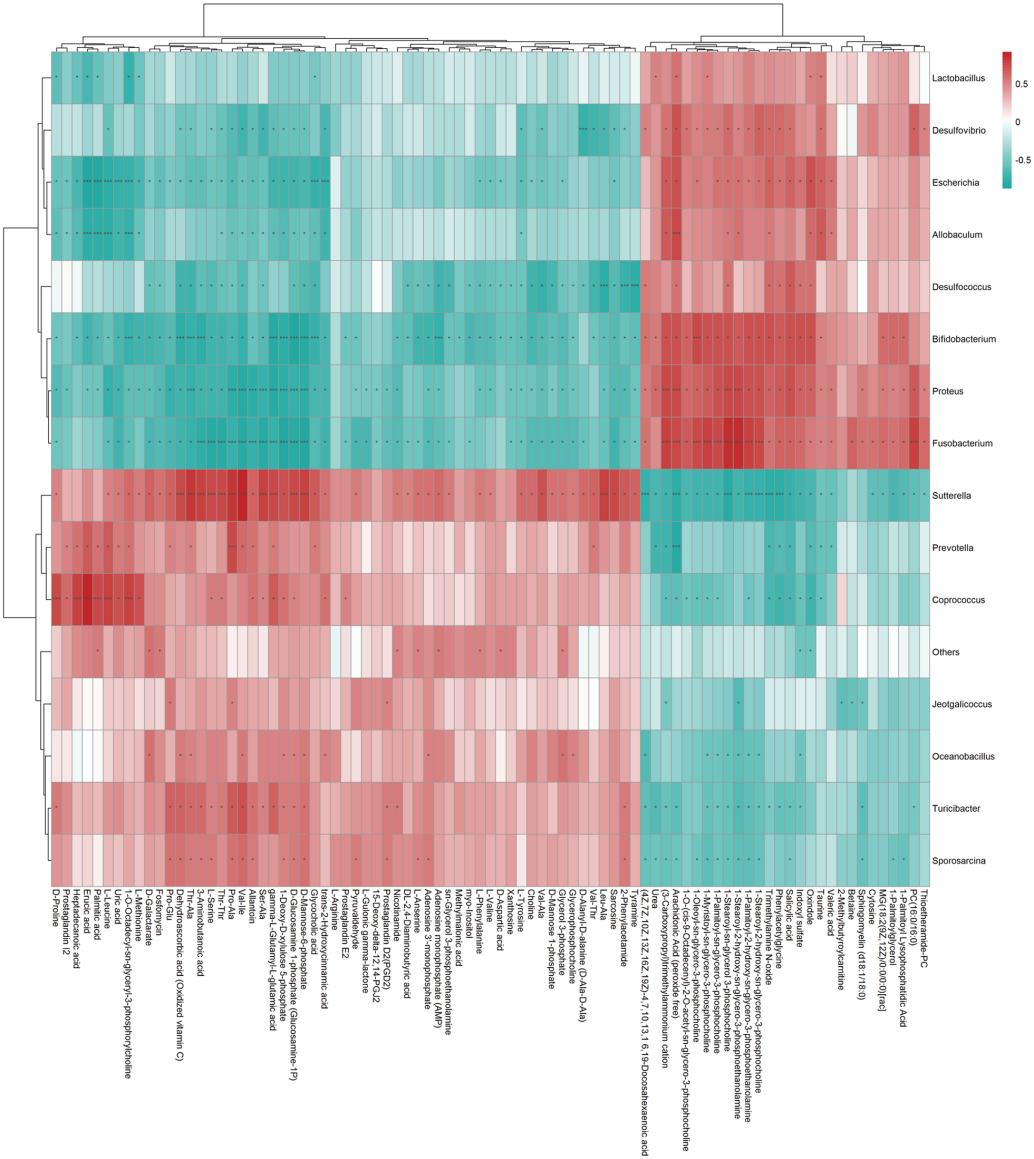
Spearman correlation analysis hierarchical clustering heat map of significantly different intestinal microbiota and significant difference metabolites. Each grid in the figure contains information on the correlation coefficient r and *p*-value, and the correlation coefficient r is represented by color. r > 0 means positive correlation, which is expressed in red, and r < 0 indicates a negative correlation, which is expressed in blue. The darker the color, the stronger the correlation. *P*-value reflects the significant level of correlation. *P*-value reflects the significance of the correlation, *0.01 < *p* < 0.05; **p < 0.01

Notably, we identified strong correlations were noted for the threshold of | r | < 1. The correlation matrix indicated correlations between the gut microbiota and the gastric tissue metabolic phenotypes (Fig. 12). Each row of the heat map represents a significantly different genus, and each column represents a significantly different metabolite. The tree branch on the left represents the results of clustering among the different genera, and the upper tree branch represents the results of the cluster analysis of the different metabolites. Clusters appearing in the same cluster with significantly different metabolites or different bacterial genera followed have similar correlation patterns.

**Fig. 13:**
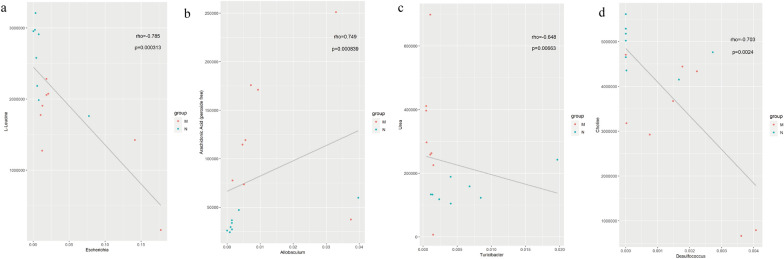
Scatter plot indicating the association between specific gut microbes and metabolites. The scattered points in the figure represent samples; rho is the spearman correlation coefficient between the relative abundance of the strain and the metabolite intensity value, and the *p*-value is the significant level of rho. **a** *Escherichia* is negatively related to l-leucine. **b** *Allobaculum* is positively related to arachidonic acid. **c** *Turicibacter* is negatively related to urea. **d** *Desulfococcus* is negatively related to choline

Unlike the spearman correlation analysis hierarchical clustering heat map, the scatter plot reflected the correlation between a single significantly different metabolite and a significantly different genus. Figure 13 depicts several typical gut intestinal microbiota-associated metabolites that were highly associated with specific intestinal bacteria to demonstrate the functional correlation between intestinal microbiota and metabolites.

## Discussion

This study explored the therapeutic mechanism of HZJD on CAG rats. In rats, CAG caused the gastric mucosa to turn white, disordered gland arrangement, and inflammatory cell infiltration. Following HZJD treatment, the pathological performance was improved, demonstrating that HZJD had a therapeutic effect on CAG (Fig. 1). Next, we performed LC–MS and 16S rRNA gene sequencing to probe the effect of HZJD on its metabolites and the intestinal microbiome of CAG rats. The data clearly showed that HZJD had a therapeutic effect on the metabolites and intestinal microbiota of CAG rats (Figs. 3, 4, 5, 6, 7, 8, 9, 10 and 11). In addition, spearman analysis showed that these perturbed intestinal microbiota were strongly associated with changes in several related metabolites (Figs. 12 and 13). Accumulating evidence has shown that metabolites related to gut microbes under drug intervention are important factors for regulating tissue function and improving health [26, 27]; thus, the mechanism of action of HZJD treatment of CAG might be due to regulation of the perturbed intestinal microbiota and its metabolites. These findings may provide mechanistic insights into HZJD treatment of CAG. In the present study, we identified 68 metabolites associated with CAG in rat gastric mucosal tissues by LC–MS (Fig. 6). The results showed that HZJD had a therapeutic effect against the alterations of 21 metabolites induced by CAG (such as choline, l-leucine, and l-serine) (Fig. 7). These affected metabolites were found to be involved in the central carbon metabolism in cancer, the mTOR signaling pathway, and the choline metabolism in cancer (Fig. 8). The 16S rRNA gene sequencing results demonstrated that CAG caused changes in the microbiota composition and relative abundance of the intestinal microbiota. Following HZJD treatment, at the genus level, the relative abundance of *Turicibacte* increased, *Desulfococcus* and *Escherichina* decreased in the CM group compared with the M group (Fig. 11).

Amino acids play an important role in regulating the energy balance in organisms, and the central carbon metabolism, namely energy metabolism, is the main source of energy for organisms. Leucine is a branched-chain amino acid that is involved in energy metabolism in the body, and plays an important role in inflammatory reactions and autophagy. The glycine, serine and threonine metabolism provides precursors for the tricarboxylic acid (TCA) cycle, all of which are involved in energy metabolism. In the present study, the levels of l-leucine and l-serine decreased in the M group compared with those in the N group, indicating that CAG energy was insufficient, which was consistent with the study by YueTao Liu et al. [17]. Previous studies have shown that there is an insufficient cellular energy supply in CAG and that decreased cellular energy metabolism may be one of the causes of gastric mucosal atrophy [28, 29]. Following HZJD treatment, l-leucine and l-serine increased in the CM group compared with the M group, suggesting that HZJD may mediated its effects in CAG by supplementing energy for the gastric mucosa.

Intestinal bacteria can affect the health of the host by regulating amino acids [28]. Evidence has shown that changes in intestinal microbes affect the utilization of amino acids, and microbes were the initial step of amino acid catabolism [30]. In the present study, l-leucine exhibited a negative correlation with *Escherichia* (Figs. 12 and 13). Leucine is one of the essential amino acids, and it plays an important role in the inflammatory response [31], *Escherichia* is one of the most common causes of bacterial infections in humans and animals and also is the primary cause of gastroenteritis [32]. Previous studies have demonstrated that l=leucine was the most effective amino acid at excluding *Escherichia* in a microfluidic assay and its concentration was inversely proportional to the number of *Escherichia* bacteria [33, 34], which is consistent with the results of this study. In the current study, l-leucine was decreased and the relative abundance of *Escherichia* was increased in the M group compared with the N group. Following HZJD treatment, the concentration of l-leucine and the relative abundance of *Escherichia* returned to normal levels, indicating that the therapeutic effect of HZJD on CAG may be achieved by reduce the abundance of *Escherichia* and increasing the l-leucine levels to provide energy for cells.

Autophagy also plays an important role in CAG, and enhanced autophagy may be one of the mechanisms leading to gastric precancerous lesions [35]. The effect of amino acids on autophagy is mediated by the mTOR signaling pathway, and l-leucine is an important nutritional signaling molecule that regulates mTOR [36]. Autophagy is pivotal for the maintenance of intestinal homeostasis, and an increase in the number of *Escherichia* bacteria has been shown to decrease the levels of autophagy [37, 38]. mTOR is the core protein involved in the regulation of autophagy, and *Escherichia* regulation of l-leucine also may be mediated by the mTOR signaling pathway, which, in turn, also may affect autophagy. However, the mechanism remains to be examined in future studies.

Microbial infections is one of the causes of CAG [4], in which the imbalance of intestinal microbiota and inflammation function together to cause damage to the mucosa [30]. CAG is a multistep, multifactor, and continuous process of inflammation [25], and persistent inflammation in the stomach is one of the important causes of intestinal microbial disorders. Arachidonic acid is an unsaturated fatty acid that is esterified in the cell membrane in the form of phospholipids. It is produced by inflammation-stimulated phospholipase and plays an important role in gastrointestinal function [39]. We previously have shown that arachidonic acid is positively correlated with *Allobaculum* (Figs. 12 and 13). *Allobaculum* is dominant in the gastrointestinal tract, and is closely related to inflammation [40]. Evidence has shown that increases in the abundance of *Allobaculum* may be an indicator of cancer [41, 42]. It is also known that microorganisms in the gut have lipases that can degrade phospholipids into polar head groups and free lipids [28]. *Allobaculum* may degrade arachidonic acid into free arachidonic acid, and although the mechanism remains unclear, current research suggests that inflammation may be the link between them. The increased abundance of *Allobaculum* increases the level of gastrointestinal inflammation, and the level of arachidonic acid increases under inflammatory conditions. Following HZJD treatment, the abundance of *Allobaculum* and arachidonic acid levels decreased, suggesting that HZJD could reduce the abundance of *Allobaculum*, inhibit inflammation, and thereby reduce the level of arachidonic acid. *Turicibacter*, an important member of the gut microbiota, is considered to be a healthy bacterial genus with anti-inflammatory effects, and may provide evidence that HZJD regulates intestinal microbiota and inhibits inflammation [31–33]. Microbes can break down urea through urease, and elevated urea indicates inflammation and infection in the body [43]. In the present study, *Turicibacter* was negatively correlated with urea (Figs. 12 and 13), in that the abundance of *Turicibacter* decreased and urea levels increased in the M group compared with N group. Following HZJD treatment, both showed a recovery trend, indicating that the HZJD could increase the abundance of *Turicibacter*, improve the anti-inflammatory ability of the gastrointestinal tract, and inhibit the development of CAG.

The use of HZJD also can changes the concentrations of some compounds participating in choline metabolism in cancer, such as choline, phosphatidylcholine (PC), and glycerophosphocholine glycerol-3-phosphate. Choline and phosphorylcholine are important components of phospholipids that are essential for the stability and integrity of cell membranes, and choline deficiency may cause gastric mucosal damage similar to CAG [44]. The decrease in choline in the M group proved that gastric mucosal cells were damaged [16]. The level of choline increased compared with the M group after the intervention of HZJD, suggesting that HZJD could increase choline levels to protect the gastric mucosa. Choline is metabolized by the intestinal microbiota and enters the body to be converted into trimethylammonium, which has been confirmed to have a role in inflammatory diseases [45]. Interestingly, *Desulfococcus* exhibited a positive correlation with trimethylamine, and a negative correlation with choline in the current study (Fig. 12). *Desulfococcus* is the dominant microbiota of sulphate-reducing bacteria, which can reduce sulfate to produce H2S, which can poison intestinal epithelial cells. In the present study, compared with the N group, the abundance of *Desulfococcus* increased in the M group, the choline level decreased in the M group, and the trimethylammonium increased in the M group. After treatment with HZJD, however, there was a trend of recovery. These results showed that HZJD could reduce the metabolism of choline to trimethylammonium by regulating the intestinal microbiota, inhibiting inflammation, and protecting the gastric mucosa. Furthermore, these findings indicated that HZJD can treat CAG by regulating *Desulfococcus* to interfere with choline metabolism.

In summary, we combined LC–MS and 16S rRNA gene sequencing to assess the impact of HZJD on the gut microbiome and its metabolic profiles in CAG rats. We used spearman analysis to calculate the correlation coefficient between the significantly different intestinal microbiota and the significantly different metabolites. Metabolomic analysis revealed that HZJD altered a variety of metabolites, which revealed its role in the central carbon metabolism in cancer, the mTOR signaling pathway, and the choline metabolism in cancer. 16S rRNA gene sequencing indicated that HZJD could regulate the diversity, microbial composition, and abundance of the intestinal microbiota of CAG rats. Spearman analysis revealed that perturbed intestinal microbiota had a strong correlation with differential metabolites, *Escherichia* exhibited a negative correlation with L-Leucine, *Turicibacter* was negatively correlated with urea, and *Desulfococcus* exhibited a positive correlation with trimethylamine, and a negative correlation with choline, and HZJD could protect CAG by increasing the abundance of *Turicibacter*, reducing the abundance of *Desulfococcus* and *Escherichia* affecting the level of related metabolites.

## Conclusions

HZJD could protect CAG by regulating intestinal microbiota and its metabolites.

## Data Availability

All data included in this article are available from the corresponding author upon request.

## Abbreviations

CAG: Chronic atrophic gastritis
HZJD: Hua-Zhuo-Jie-Du
CM: Chinese medicine
M: Model
N: Normal
SD: Sprague–Dawley
H&E: Hematoxylin and eosin staining
QC: Quality control
LC–MS: Liquid chromatograph mass spectrometer
HPLC: High performance liquid chromatography
HILIC: Hydrop interaction liquid chromatography
ESI: Electrospray ionization
RPLC: Reversed-phase liquid chromatography
OTUs: Operational taxonomic units
KEGG: Kyoto encyclopedia of genes and genomes
PCA: Principal component analysis
OPLS-DA: Orthogonal par-tial least square-discriminate analysis
FC analyisis: Fold change analysis
PCoA: Principal coordinates analysis
LDA: Linear discriminant analysis
TCA: Tricarboxylic acid
